# Synopsis of Human Anatomy

**Published:** 1889-08

**Authors:** 


**Synopsis of Human Anatomy**, being a complete compend of Anatomy, including the Anatomy of the Viscera, and numerous tables. By JAMES K. YOUNG, M. D., Instructor in Orthopedic Surgery and Assistant Demonstrator of Surgery in the University of Pennsylvania; Fellow of the College of Physicians; etc., etc. 12mo, cloth, pp. ix., 393. Philadelphia and London: F. A. Davis, publisher. 1889.

Dr. Young has compiled a very useful book. We are not inclined to approve of compends as a general rule, but it certainly serves a good purpose to have the subject of anatomy presented in a compact, reliable way, and in a book easily carried to the dissecting-room. This the author has done. The book is well printed, the illustrations well selected, and the text judiciously chosen. If a student can indulge in more than one work on anatomy,—for, of course, he must have a general treatise on the subject,—he can hardly do better than to purchase this compend. It will save the larger work, and can always be with him during the hours of dissection. The book appears as one of the Physicians' and Students' Ready Reference series published by Mr. F. A. Davis, and we believe will serve the purpose for which it was intended. F. H. P.

